# The double burden of disease and the challenge of health access: Evidence from Access, Bottlenecks, Cost and Equity facility survey in Ghana

**DOI:** 10.1371/journal.pone.0194677

**Published:** 2018-03-23

**Authors:** Mawuli Komla Kushitor, Sandra Boatemaa

**Affiliations:** Regional Institute for Population Studies, University of Ghana, Legon, Accra, Ghana; Harvard Medical School, UNITED STATES

## Abstract

Despite the double burden of infectious and chronic non-communicable diseases in Africa, health care expenditure disproportionately favours infectious diseases. In this paper, we examine quantitatively the extent of this disproportionate access to diagnoses and treatment of diabetes, hypertension and malaria in Ghana. A total of 220 health facilities was surveyed across the country in 2011. Findings indicate that diagnoses and treatment of infectious diseases were more accessible than NCDs. In terms of treatment, 78% and 87% of health facilities had two of the recommended malaria drugs while less than 35% had essential diabetes and hypertension drugs. There is a significant unmet need for diagnoses and treatment of NCDs in Ghana. These inequities have implications for high morbidity and mortality from NCDs. We recommend the use of task shifting as a model to increase the delivery of NCD services.

## Introduction

Africa, is experiencing a double burden of diseases [1]. This is characterised by the increasing prevalence of chronic non-communicable diseases (NCD) and the battle to deal with infectious diseases [2,3]. The burden of NCDs disproportionately affects populations in low and middle income countries (LMIC) where health systems are weak [4]. In LMICs, especially in sub Saharan Africa (SSA), hypertension is increasing rapidly because of rapid population growth, increased life expectancy and lifestyle factors [5–7]. Age specific prevalence rates of hypertension are highest in SSA (46%) and lowest in the America’s (36%) [4].

In Ghana, the prevalence of NCDs such as obesity, hypertension, diabetes and heart diseases have been rising [8–10]. In 2013, NCDs contributed 22.2% of deaths at the Korle-Bu Teaching Hospital [11]. The Ghana Ministry of Health (MoH) 2010 annual report indicated that hypertension is the second leading cause of outpatient morbidity in adults 45 years and above [12].

Research, however, shows that adequate blood pressure treatment and control can significantly reduce the first incidence of heart attack, strokes and recurrent strokes, heart failure, chronic kidney disease and premature death [13,14]. However, limited access to health care is a barrier to preventing the epidemic of hypertension, diabetes and other cardiovascular diseases in the country is experiencing [15,16]. There is a low level of awareness of hypertension and diabetes [17–19]. In Africa, less than 30% of people living with hypertension are on biomedical treatment [20,21]. The majority of people living with hypertension and diabetes are not on treatment because of limited access and cost of biomedical treatment [22]. As a result, people living with NCDs healer shop which increases the risk of complications and mortality [23].

In this paper, we examine quantitatively the extent of this disproportionate access to diagnoses and treatment of diabetes, hypertension and malaria in Ghana. These disease conditions were selected because they are the leading causes of morbidity and deaths among the Ghanaian population [11,24,25]. This paper focuses on addressing access to health care because social organisations and processes at the macro level generate health inequalities. Understanding the inequalities in health and the policies which might reduce these is essential for addressing the cardiovascular public health challenge Africa faces.

## Materials and methods

Data from the Access, Bottlenecks, Costs and Equity (ABCE) Facility Survey, Ghana was used. The survey was part of a project jointly conducted by the Ministry of Health, Ghana (MoH), Institute for Health Metrics and Evaluation (IHME), Ghana Health Service (GHS), and UNICEF [26]. The Ghana Health Service provided ethical clearance for the project. A nationally representative sample of health facilities in Ghana were selected. The data was collected between June and October 2012. The questionnaire was administered to facility administrators. A two-stage stratified sampling design was used to select rural and urban districts as identified by the 2011 Multiple Indicator Cluster Survey (MICS). In the first sampling stage one rural and one urban district was randomly selected from the ten regions of Ghana (n = 20). In addition, the Accra and Kumasi metropolitan areas were purposively added to the selected districts [26].

The second stage of sampling facilities took place across a range of platforms. The ABCE project defined a platform as the channel used to deliver health services. In Ghana 9 platforms were identified. These were: 1) teaching hospitals; 2) regional referral hospitals; 3) hospitals; 4) health centres; 5) Community-Based Health Planning and Services (CHPS), 6) maternity clinics 7) pharmacies and 8) drug stores, as well as District Health Monitoring Teams (DHMT). A facility sampling frame was developed based on 2011 MoH Needs Assessment. In each district 4 hospitals, health centres, CHPS and maternity clinics were sampled. In addition, two pharmacies were sampled. Out of the 240 facilities sampled, 220 responded. The survey response rate was 92%.

### Measurement of variables

The provision of diagnostic and treatment services for malaria, hypertension and diabetes were the main outcome variables. The dataset has information on the type of services offered by health facilities from 2007 to 2011 [26]. In 2010, Ghana implemented the test-before treatment policy for malaria. As a result of this policy, healthcare providers are expected to have Rapid Diagnostic Test (RDT) [27]. A facility was classified as providing malaria test if they had the RDT available at the time of the survey. Also in 2011, health facilities were expected as part of health procedures to provide blood pressure measurements for all out-patient clients. A health facility was classified as providing hypertension diagnosis if they had blood pressure apparatus. The fasting blood glucose test was the measure used to assess diabetes diagnosis. Facilities that reported providing fasting blood glucose were classified as providers of diabetes diagnosis.

To pharmacologically treat diabetes among obese individuals with Type 2 diabetes the MoH recommends Metformin and/or Thiazolidinediones [28]. Among patients with ketoacidosis, Type 1 diabetes, in pregnant and breast- feeding women, whether Type 1 or Type 2, and Type 2 patients insulin is recommended. Diabetes treatment at the facility level was measured with the availability of Metformin Tablet, 500 mg and insulin. The pharmacological treatment recommended for hypertension includes Beta-blockers (e.g Atenolol, oral, 50–100 mg daily) and Thiazide diuretics (e.g., Bendroflumethiazide (bendrofluazide) oral, 2.5 mg daily) [28]. In the ABCE survey, facilities were asked to indicate whether they had Atenolol. Therefore, facilities which said yes were classified as providing hypertension treatment. To treat uncomplicated malaria the MoH recommends Artesunate-Amodiaquine, Artesunate-Lumefantrine, or Dihydroartemisinin-Piperaquine oral tablets while quinine is recommended for treatment of severe malaria. Facilities were classified as providers of malaria treatment if they had Artesunate-Amodiaquine and Quinine at the time of survey.

We used five facility-level variables from the ABCE dataset as independent variables: type of facility, facility years, type of facility management, location of facility, and region of location. The type of facility was measured using the platform created by the GHS as a categorical variable: CHPS, health centre, hospital, maternity home, pharmacy, private clinic and referral hospital. The facility years refer to the number of years the facility has been delivering health services. It was measured as a categorical variable (1–5 years, 6–10 years, 11–15 years, 16–20 years and 21+ years). The type of management of the facility was classified as public or private. Facilities that were owned by the government of Ghana or a mission, but managed by the MoH were listed as public facilities while facilities owned by a mission (but not managed by the MoH) or private enterprise were categorised as private. The geographical location of the facilities was measured by the ten regions of the country and whether the facility is located in an urban or rural area.

### Analysis

All analyses were carried out using the STATA statistical software package version 12 (2017; StataCorp, College Station, TX, USA). The data were used without weighting. In the data release information sheet all the factors needed to generate weight were not provided [26]. Although, the data were not weighted, the sample is representative of the types of health facilities in Ghana due to the facility sampling frame used. In each district, four hospitals, health centre, CHPS, maternity clinics and two pharmacies were selected [26]. Using ABCE data, the proportions of the characteristics of facilities and the services they provided were calculated. The Pearson’s chi-squared test statistic was calculated to test for differences in proportions. A trend analysis was done for the treatment and testing procedures from 2007 to 2011. The association between facility characteristics and service delivery were analysed for the year 2011 only.

## Results

### Characteristics of facilities

In 2011, 220 of the facilities were operational. Among them, 29% were CHPS compound and 20% were health centres. Table 1 indicates that about 50% of the facilities were in rural areas while only 15% and 34% were in peri-urban and urban areas, respectively. The data show that 12% of the facilities were in the Ashanti region, and a tenth in Central, Eastern, Greater Accra, Northern, Upper West and Western regions. The proportion of facilities managed by the government was 59%.

**Table 1:** Characteristics of health facities in 2011.

Variable	Frequency	%
**Platform**		
CHPS	65	29.6
Health centre	43	19.6
Hopsital	18	8.2
Maternity home	16	7.3
Pharmacy	37	16.8
Provate clinic	30	13.6
Referral hospital	11	5.0
**Location**		
Rural	109	49.5
Peri/Semi-urban	35	15.9
Urban	76	34.5
**Region of facility**		
Ashanti	26	12.4
Brong-Ahafo	17	8.1
Central	21	10.1
Eastern	22	10.5
Greater Accra	21	10.1
Northern	21	10.1
Upper East	20	9.6
Upper West	22	10.5
Volta	18	8.6
Western	21	10.1
**Management type**		
Public	129	58.6
Private	91	41.4
**Facility years**		
1–5	62	28.2
6–10	43	19.6
11–15	26	11.8
16–20	26	11.8
21+	63	28.6
**Total**	**220**	**100.0**

### Diagnosis of malaria, hypertension and diabetes

Table 2 present the trend analysis of facilities from 2007 to 2011. The results show that while the BP monitors were present at most facilities there are huge variations in the availability of RDT and FGB test. The proportion of facilities with BP monitors has remained relatively stable over the period from 88% in 2007 to 86% in 2011. The proportion of facilities providing RDT services increased from 45% in 2009 to 54% in 2010-an increase of 20% over the period (Table 2). There was a decline in the proportion of facilities providing fasting blood glucose test between 2008 and 2009, however, this increase was short lived as the proportion reduced by 8% in 2011 (Table 2). Between 2007 and 2008, there was an 8% reduction in the facilities providing blood glucose test. The findings indicate that while RDT kits and BP monitors have been relatively stable at facilities, diabetes diagnosis equipment is declining.

**Table 2:** Trends in RDT, fasting blood glucose and blood pressure diagnosis services at health facilities in Ghana, 2007–2011.

Type of service	RDT		Fasting blood glucose		Blood pressure monitor	
Year	Facilities providing service (%)	% Change	Facilities providing service (%)	% Change	Facilities providing service (%)	% Change
2007	47		38		88	
2008	46	-2.2	37	-2.6	86	-2.3
2009	45	-2.2	36	-2.7	86	0.0
2010	54	20.0	37	2.8	86	0.0
2011	54	0.0	34	-8.1	86	0.0

Table 3 present findings on the availability of diagnostic services by facility characteristics in 2011. The availability of RDT kits significantly differed by facility type (p = 0.000). All the hospitals and referral hospitals had RDT kits (100%). The pharmacy was the outlet with the least proportion of outlets with the RDT kits (3%). A significantly higher proportion of the urban facilities had RDT kits (62%) compared to rural and peri-urban centres (35% and 40%, respectively). The Eastern, Upper West and Central regions, were the regions with most of their health facilities without RDT kits. Less than 25% of the facilities in these regions had RDT kits. There was no significant difference in the availability of RDT kits by management type. The availability of RDT kits significantly fluctuates with years of service. About a one-third of facilities that are have been operational for 1–5 years had RDT kits while only 9% of those 6–10 years had RDT kits.

**Table 3:** Availability of RDT, glucometer and blood pressure monitor by facility characteristics, 2011.

Variable	RDT(%)	Glucometer (%)	BPM (%)	N
**Platform**				
CHPS	23.1	6.0	98.5	65
Health centre	53.5	34.8	97.7	43
Hospital	100.0	100.0	100.0	18
Maternity home	50.0	43.7	100.0	16
Pharmacy	2.7	2.7	22.2	37
Private clinic	80.0	80.0	100.0	30
Referral hospital	100.0	100.0	100.0	11
*P-value*	0.0	0.0	0.0	
**Location**				
Rural	35.8	18.3	88.1	109
Peri/Semi-urban	40.0	40.0	74.3	35
Urban	61.8	60.5	89.3	76
*P-value*	0.002	0.000	0.076	
**Region**				
Ashanti	65.4	61.5	80.8	26
Brong-Ahafo	47.1	52.9	76.5	17
Central	23.8	19.0	85.7	21
Eastern	13.6	13.6	81.8	22
Greater Accra	80.9	66.7	100.0	21
Northern	47.6	33.3	85.7	21
Upper East	45.0	20.0	85.0	20
Upper West	22.7	22.7	86.4	22
Volta	33.3	16.7	94.1	18
Western	42.8	19.0	80.9	21
*P-value*	0.000	0.000	0.657	
**Management type**				
Public	43.9	41.8	68.9	129
Private	46.5	32.6	98.3	91
*P-value*	0.708	0.162	0.000	
**Facility years**				
1–5	35.5	24.2	88.7	62
6–10	20.9	16.3	78.6	42
11–15	53.8	42.3	84.6	26
16–20	53.8	42.3	88.5	26
21+	65.1	57.1	88.9	63
*P-value*	0.000	0.000	0.566	** **
**Total**	**45.4**	**36.3**	**86.3**	**220**

The availability of FBG test differed significantly by type of facility. FBG test at pharmacies and CHPS compound was very limited, only 3% and 6% of these facilities had the service available in 2011, respectively. About a two-fifths of the maternity homes had the FBG test available. A higher proportion of rural facilities reported the lack of glucometers compared to urban facilities. For example, 18% of rural facilities had FBG test available compared to 60% of urban facilities. In four regions, less than 20% of their facilities had FBG test available. The Ashanti and Greater Accra regions were the only regions with about 60% of their facilities with glucometers. Facilities that had been operational for 6–10 years had the least proportion of centres with glucometers.

Blood pressure monitors were available at almost all facilities except pharmacies. The pharmacies had the least share of facilities with BP monitors (20%). The availability of BP monitors at health facilities did not differ significantly by location and region. Almost 80% of all the facilities in each region had BP monitors. A significantly higher proportion of private facilities had BP monitors compared to the public facilities (98% versus 69%). Facility years did not influence the availability of BP monitors.

### Treatment of malaria, hypertension and diabetes

Table 4 compares treatment trends from reports obtained in 2007, 2008, 2009, and 2010 with information gathered in the 2011. Across the years the proportion of facilities that provided malarial treatment were significantly higher than those who provided diabetes and hypertension treatment. For example, while the proportion of facilities with Artesunate-Amodiaquine has been relatively stable over the period, the proportion of facilities with insulin has been declining (Artesunate-Amodiaquine79% to 78%, versus Insulin 23% to 20%). Similarly, another decrease of 2% was also reported for nifidepine between 2010 and 2011. In terms of hypertension treatment, the availability of Atenol has remained at 33% since 2009.

**Table 4:** Trends in malaria, hypertension and diabetes treatment services at health facilities in Ghana, 2007–2011.

Health condition	Malaria				Hypertension				Diabetes			
Type of treatment	Quinine		Artesonote-Amodiaquine		Nifedine		Atenol		Metformin		Insulin	
Year	Facilities providing service (%)	% Change	Facilities providing service (%)	% Change	Facilities providing service (%)	% Change	Facilities providing service (%)	% Change	Facilities providing service (%)	% Change	Facilities providing service (%)	% Change
2007	64		79		55		35		38		23	
2008	63	-1.6	78	-1.3	53	-3.6	34	-2.9	36	-5.3	23	0.0
2009	61	-3.2	78	0.0	51	-3.8	33	-2.9	34	-5.6	21	-8.7
2010	61	0.0	78	0.0	51	0.0	33	0.0	34	0.0	21	0.0
2011	60	-1.6	78	0.0	50	-2.0	33	0.0	34	0.0	20	-4.8

Table 5 shows the availability of treatment in 2011 by facility characteristics. Most of the facilities had malaria treatment compared to diabetes and hypertension. Ninety-one percent of referral hospitals had Artesunate-Amodiaquine. This proportion declined to 70% at pharmacies and private hospitals. In addition, all the hospitals had Quinine for the treatment of severe malaria. The availability of Artesunate-Amodiaquine did not differ significantly, by location and region of the facility.

**Table 5:** Treatment of malaria, hypertension and diabetes by facility characteristics, 2011.

Variable	Malaria (%)		Hypertension (%)		Diabetes (%)	
	Artesonate	Quinine	Atenol	Nifedine	Insulin	Metformin
**Platform**						
CHPS	75.4	27.7	4.6	9.2	0.0	0.0
Health centre	88.4	83.7	13.9	53.5	2.3	13.9
Hopsital	88.9	100.0	83.3	94.4	72.2	100.0
Maternity home	75.0	75.0	12.5	68.7	6.2	18.7
Pharmacy	70.3	51.3	40.5	52.8	29.7	40.5
Provate clinic	70.0	63.3	70.0	80.0	30.0	76.7
Referral hospital	90.9	90.9	90.9	90.9	90.9	90.9
*P-value*	0.253	0.000	0.000	0.000	0.000	0.000
**Location**						
Rural	80.7	48.6	10.1	30.3	2.7	11.0
Peri/Semi-urban	85.7	60.0	42.8	47.1	22.9	40.0
Urban	71.1	76.3	60.5	80.3	44.7	64.5
*P-value*	0.146	0.001	0.000	0.000	0.000	0.000
**Region of residence**						
Ashanti	57.7	65.4	23.1	56.0	15.4	50.0
Brong-Ahafo	88.2	76.5	52.9	52.9	29.4	47.1
Central	20.9	42.8	14.3	33.3	14.3	19.0
Eastern	68.2	27.2	22.7	31.8	13.6	18.2
Greater Accra	80.9	71.4	71.4	76.2	33.3	61.9
Northern	66.7	66.7	42.9	52.4	14.3	33.0
Upper East	85.0	60.0	20.2	60.0	0.0	10.0
Upper West	86.4	68.2	18.2	40.9	18.2	27.3
Volta	77.8	50.0	27.8	44.4	22.2	27.8
Western	90.5	57.1	9.5	33.3	9.5	14.3
*P-value*	0.144	0.046	0.000	0.084	0.000	0.002
**Management type**						
Public	83.7	58.1	24.0	38.8	25.3	50.5
Private	70.3	62.6	45.0	66.7	17.0	22.8
*P-value*	0.018	0.502	0.001	0.000	0.137	0.000
**Facility years**						
1–5	71.0	45.2	17.7	33.9	8.1	16.1
6–10	90.7	58.1	25.6	47.6	18.6	20.9
11–15	84.6	69.2	38.5	65.4	19.2	42.3
16–20	73.1	73.1	30.8	50.0	15.4	38.5
21+	76.2	66.7	50.8	61.9	36.5	55.6
*P-value*	0.135	0.046	0.002	0.014	0.003	0.000
**Total**	**78.2**	**60.0**	**32.7**	**50.2**	**20.4**	**34.1**

The results show that a higher proportion of facilities had Nifidipine (50%) than Atenol (33%). The availability of hypertension medicines was highly limited at the CHPS compounds. For example, less than 10% of the CHPS facilities had Atenol and Nefidipine (p = 0.000). A higher proportion of the urban and peri urban health facilities had hypertension drugs compared to the rural facilities. For example, while 60% of urban facilities had Atenol only 10% of the rural facilities had the drug at the time of the survey. More treatment medications were available at private facilities than at public facilities.

Diabetes medications were not observed at the CHPS compounds. The treatment of diabetes was limited in the rural areas compared to the urban areas (p = 0.000). Less than 10% of the rural facilities had Metformin and Insulin. The Upper East, Western Region and Central regions had very limited diabetes medications. None of the Upper East facilities and less than 10% of the facilities in the Western region had metformin.

## Discussion

This paper explored the inequalities in the diagnosis and treatment of NCDs compared to infectious diseases. While several studies have linked the poor control of NCDs to limited NCD care, we provide empirical evidence to show the inequalities in the allocation of health resources for the management of NCDs. We find that a higher proportion of health facilities provide malaria diagnosis and treatment services compared to hypertension and diabetes care. Diabetes treatment is the least provided for amongst the health facilities across the country. The longitudinal findings indicate that the inequalities has persisted since 2007. Diabetes and hypertension treatment are almost absent at CHPS facilities and health centres. We provide two explanations for our findings.

First, there is lower health spending in Ghana. According to the Abuja Declaration, governments are expected to allocate 15% of the total national budget to health care [29]. However, since Ghana signed onto the declaration, the percent of the national budget allocated to health has never been up to 15%. Actually, the national allocation has declined from 13.5% in 2012 to 9.5% in 2015 and 13% in 2017 [30,31]. This has reflected in a reduction of percentage share of GDP on health from 5.3 in 2007 to 3.6 in 2014 [32]. In addition to the limited health budget, health expenditure disproportionately favours infectious diseases at the expense of the rising burden of NCDs in the country [33,34].

Secondly, some facilities do not have the mandate to provide certain services. For example, the specific elements of service delivery of the CHPS facility prioritise immunization, maternal and child health, and family planning services [35,36]. As a result, these facilities are not provided with the infrastructure and supplies that would enable them deliver NCD care services. In addition, personnel at these facilities are not trained to deliver NCD care.

In order to address this challenge, this study recommends task shifting of NCD care. Task shifting is the defined as the rational distribution of task amongst a health workforce team [37]. There is mounting evidence that nurses are equally effective in transmitting and providing basic NCD care if given the needed training [37–40]. Specifically, in the case of uncomplicated hypertension and diabetes, studies show that risk assessment and management of risk factors can be carried out by trained nurses. Given that, this study shows limited resources available for people living with diabetes and hypertension within the formal health care system, a task shifting approach can potentially widen the reach of basic hypertension and diabetes management to the remotest areas in Ghana. We suggest that basic NCD care should be layered onto the existing CHPS services. Currently, the CHPS service is focused on providing antenatal, post-natal and child health services. The salaried community health nurses who are deployed to village locations to can be trained through task shifting to also provide some uncomplicated NCD services.

We believe adding basic NCD care that is focused on hypertension and diabetes is a cost effective approach that can potentially improve the control of hypertension and diabetes within communities in Ghana. Furthermore, research shows that primary and secondary prevention of uncomplicated hypertension management involves significant lifestyle changes and standard pharmacotherapy, which can be delivered by trained nurses [37]. Already, some scholars have successfully piloted CVD care using the CHPS process. In summary, task shifting can be used to reduce the impact of insufficient health personnel, a major cause of service unavailability in health facilities [41].

The major limitation of this study is that the data is seven years old and may not reflect current situations at health facilities. However, we used this data because it is the only comprehensive national dataset that provides information on health facilities [26]. Secondly, the conditions in the facilities may not have changed much because the percent spending on health has actually declined since 2011 [30,31,42].

## Conclusion

This study examined the unequal provision of NCD diagnosis and treatment compared to infectious diseases at health facilities in Ghana. The findings indicate that there is a significant unmet need for diagnoses and treatment of hypertension and diabetes in Ghana. This paper is not prioritising NCDs over infectious diseases, but revealing at a deeper level, the limited support for NCD care in the Ghanaian health sector. The imbalance in the allocation of health care resources has implications for high morbidity and mortality from preventable causes such as hypertension and diabetes. We recommend a conscious structural effort to devote resources to the provision of NCD related services and task-shifting of NCD care.
